# ABCA1, ABCG1, and ABCG4 Are Distributed to Distinct Membrane Meso-Domains and Disturb Detergent-Resistant Domains on the Plasma Membrane

**DOI:** 10.1371/journal.pone.0109886

**Published:** 2014-10-10

**Authors:** Osamu Sano, Shiho Ito, Reiko Kato, Yuji Shimizu, Aya Kobayashi, Yasuhisa Kimura, Noriyuki Kioka, Kentaro Hanada, Kazumitsu Ueda, Michinori Matsuo

**Affiliations:** 1 Division of Applied Life Sciences, Graduate School of Agriculture, Kyoto University, Sakyo, Kyoto, Japan; 2 Department of Biochemistry and Cell Biology, National Institute of Infectious Diseases, Tokyo, Japan; 3 Institute for Integrated Cell-Material Sciences (iCeMS), Kyoto University, Sakyo, Kyoto, Japan; 4 Department of Food and Nutrition, Faculty of Home Economics, Kyoto Women’s University, Kyoto, Japan; University of Cambridge, United Kingdom

## Abstract

ATP-binding cassette A1 (ABCA1), ABCG1, and ABCG4 are lipid transporters that mediate the efflux of cholesterol from cells. To analyze the characteristics of these lipid transporters, we examined and compared their distributions and lipid efflux activity on the plasma membrane. The efflux of cholesterol mediated by ABCA1 and ABCG1, but not ABCG4, was affected by a reduction of cellular sphingomyelin levels. Detergent solubility and gradient density ultracentrifugation assays indicated that ABCA1, ABCG1, and ABCG4 were distributed to domains that were solubilized by Triton X-100 and Brij 96, resistant to Triton X-100 and Brij 96, and solubilized by Triton X-100 but resistant to Brij 96, respectively. Furthermore, ABCG1, but not ABCG4, was colocalized with flotillin-1 on the plasma membrane. The amounts of cholesterol extracted by methyl-β-cyclodextrin were increased by ABCA1, ABCG1, or ABCG4, suggesting that cholesterol in non-raft domains was increased. Furthermore, ABCG1 and ABCG4 disturbed the localization of caveolin-1 to the detergent-resistant domains and the binding of cholera toxin subunit B to the plasma membrane. These results suggest that ABCA1, ABCG1, and ABCG4 are localized to distinct membrane meso-domains and disturb the meso-domain structures by reorganizing lipids on the plasma membrane; collectively, these observations may explain the different substrate profiles and lipid efflux roles of these transporters.

## Introduction

Membranes exhibit several different lipid phases, depending on their lipid compositions and temperature [1]. Typical sphingomyelin (SM), which has a hydrophobic moiety consisting of a sphingosine base with a *trans*-double bond and saturated fatty acids chain, forms stretched conformations in membrane lipid bilayers. By contrast, most natural glycerophospholipids, which have unsaturated fatty acyl chains with *cis*-double bonds, cannot form such conformations. These structural features of SM allow it to make more intimate molecular contacts with cholesterol, generating stronger van der Waals interactions, as compared to unsaturated glycerophospholipids [2]. Furthermore, the bulky phosphocholine head group of SM may contribute to shield the hydrophobic part of cholesterol molecules from water molecules under aqueous environments [3]. Consequently, SM and cholesterol tend to be closely packaged and to form membrane meso-domains with a liquid ordered phase.

Because both SM and cholesterol are abundant at the plasma membrane in mammalian cells, the SM/cholesterol-enriched meso-domains exist mainly at the plasma membrane. These SM/cholesterol-enriched meso-domains are often considered to be part of ‘lipid rafts’, although the definition of lipid rafts is still under debate [4], [5], [6]. When cells or isolated membranes are treated with several types of non-ionic detergents at low temperatures, various components associated with the SM/cholesterol-enriched meso-domains or lipid rafts are distributed to the detergent-insoluble fractions [7]. For convenience, we tentatively regard such detergent-insoluble fractions as a biochemical representative of lipid rafts in the present paper.

Several detergents, such as Triton X-100, CHAPS, Brij 96, and Lubrol WX, have been used to prepare raft domains. Among them, Brij 96 and Lubrol WX are milder than Triton X-100 for solubilizing membrane lipids [8]. Glycosylphosphatidyl inositol-anchored proteins, flotillin, and gangliosides are targeted to lipid rafts. There are specialized raft domains, caveolae, at the cell surface [6]. Caveolae are invaginations of the plasma membrane and contain polymerized caveolin, which is a hairpin-like integral membrane protein. Raft domains play important roles in membrane trafficking, substrate transport, and signal transduction including IgE receptor signaling, T-cell antigen receptor signaling, and epidermal growth factor receptor signaling [6], [9].

ATP-binding cassette G1 (ABCG1) and ABCG4 are members of the ABCG subfamily of proteins, which are half-type ABC proteins. They consist of an *N*-terminal cytosolic nucleotide-binding domain (NBD) and a *C*-terminal transmembrane domain (TMD), which has 6 transmembrane α-helices. ABCG1 and ABCG4 form a homodimer or a heterodimer [10], [11], [12], [13]. ABCG1 mediates the efflux of cholesterol, 7-ketocholesterol, SM, and phosphatidylcholine (PC) to high-density lipoprotein (HDL) from cells [10], [14], [15]. ABCG4, which shares 69% identity and 84% similarity at the amino acid level with ABCG1, mediates the efflux of cholesterol to HDL, like ABCG1 [14]. ABCG1 is ubiquitously expressed, but highly expressed in the brain, lung, and liver. ABCG4 is expressed in the eye, brain, and in bone marrow megakaryocyte progenitors. Chow-fed mice lacking Abcg1 showed accumulation of phospholipids and neutral lipids, including cholesterol and triglyceride, in liver and lung [16], [17]. Mice lacking Abcg1 and Abcg4 showed high levels of oxysterols and ketosterols in the brain, which are toxic to neurons [18]. These studies suggest that ABCG1 plays an important role in the removal of excess cholesterol from peripheral cells and that ABCG1 and ABCG4 protect cells from toxic sterols in the central nervous system. In addition to cholesterol removal from cells, ABCG1 has other physiological functions. The absence of Abcg1 in mice abolished the regulation of T-cell proliferation by liver X receptor signaling, suggesting that ABCG1 suppresses the proliferation of T cells [19]. Expression of ABCG1 induced the apoptosis of cultured cells [20], but inhibited the apoptosis of macrophages by decreasing raft-dependent signaling of TLR4 and NOX2 [21], and blocked apoptosis in prostate cancer cells by downregulating Akt signaling in raft domains [22]. Abcg4 suppresses the proliferation of megakaryocyte progenitor cells by decreasing c-MPL signaling in raft domains [23]. These findings suggest that ABCG1 and ABCG4 are involved in cell proliferation, apoptosis, and the immune response, and that these various functions may be related to the regulation of raft domains where many signaling pathways are executed. However, it is not clear if ABCG1 and ABCG4 are involved in the regulation of raft domains.

Besides ABCG1 and ABCG4, ABCA1 is also involved in cholesterol efflux from cells. ABCA1 is a member of the A subfamily of ABC proteins, which has two TMDs and two NBDs. Mutations in *ABCA1* cause a genetic disease, Tangier disease, characterized by the loss of HDL from serum [24], [25], [26]. ABCA1 is expressed ubiquitously and mediates the efflux of cholesterol and PC to apolipoprotein A-I (apoA-I), which forms preβ-HDL [27], [28]. Previous studies have shown that ABCA1 and ABCG1 or ABCG4 coordinately remove excess cholesterol from cells [29], [30]. ABCA1 disrupts raft domains, as detected by a loss of caveolin localization to raft domains, which leads to reduced Akt phosphorylation in response to signaling by epidermal growth factor [31]. ABCA1 and ABCG1 have been reported to increase the proportion of cholesterol accessible to cholesterol oxidase [32], [33], suggesting that the levels of cholesterol in non-raft domains are increased by the disruption of raft domains. These findings also raise the possibility that ABCG1 regulates raft domain structures by redistributing lipid molecules.

In the Chinese hamster ovary (CHO) mutant cell line, LY-A, ceramide transfer is impaired by a missense mutation in CERT, which transfers ceramide from the endoplasmic reticulum (ER), where it is synthesized, to the Golgi, where it is used to synthesize SM [34], [35], [36]. We have shown that the efflux of cholesterol and SM mediated by ABCG1 is impaired in LY-A cells. This suggests that raft domains are important for the function of ABCG1 because raft structures are disturbed in LY-A cells [37]. In contrast to ABCG1, the efflux of cholesterol and PC to apoA-I by ABCA1 increased in the LY-A cells as compared to controls. We and others have demonstrated that ABCA1 is localized to non-raft domains and mediates the efflux of cholesterol from non-raft domains [38], [39]. It has been reported that ABCG1 is not colocalized with caveolin-1 [40], but interacts with caveolin-1 [41]. It is, however, unclear whether ABCG1 and ABCG4 are localized to raft or non-raft domains.

In the present study, we examined and compared the distributions of ABCA1, ABCG1, and ABCG4 on the plasma membrane and investigated the functional relationships between raft domains and the activities of ABCA1, ABCG1, and ABCG4. We investigated the efflux of cholesterol by ABCG4 in LY-A cells to reveal the effect of raft structure on the function of ABCG4, and to compare the result with those for ABCG1 and ABCA1 obtained in our previous studies. We examined the localizations of ABCA1, ABCG1, and ABCG4 to membrane meso-domains by using membrane solubilization and fractionation with detergents. Furthermore, we examined the effects of ABCA1, ABCG1, and ABCG4 on raft structure. We demonstrated that they were localized to distinct membrane meso-domains, and induced the remodeling of raft domains.

## Materials and Methods

### Materials

Rabbit polyclonal anti-ABCG1 and anti-flotillin-1 antibodies were purchased from Santa Cruz (Santa Cruz, CA). Rabbit polyclonal anti-caveolin-1, mouse anti-clathrin heavy chain, and mouse anti-FAK antibodies were obtained from BD Transduction Laboratory (Lexington, KY). Mouse anti-paxillin antibody and a cholera toxin subunit B Alexa 555 conjugate were purchased from Invitrogen (Carlsbad, CA). An anti-ABCA1 antibody was provided by Kyowa Hakko Kirin Co., Ltd. (Tokyo, Japan). HDL was acquired from Calbiochem (San Diego, CA). Human embryonic kidney (HEK) 293 cells and SH-SY5Y cells were obtained from American Type Culture Collection (Manassas, VA). Other chemicals were purchased from Sigma-Aldrich (St. Louis, MO), GE Healthcare (Little Chalfont, UK), Cayman Chemical (Ann Arbor, MI), Wako Pure Chemical Industries (Osaka, Japan), and Nacalai Tesque (Kyoto, Japan).

### Preparation of antibody against the NBD of ABCG4

The NBD (amino acids 1–353) of human ABCG4 was fused with maltose-binding protein in a pMALc2 vector (New England Biolabs Inc., Ipswich, MA), and fusion protein was expressed in *Escherichia coli* strain BL21. The fusion protein was purified with amylose resin (New England Biolabs Inc.), and a rabbit polyclonal antibody was raised against the purified protein.

### Cell culture

LY-A and LY-A/CERT cells [35] were grown in Ham’s F-12 medium supplemented with 10% (v/v) fetal bovine serum (FBS) in 5% CO_2_ at 37°C. When cultured in a sphingolipid-deficient medium, cells were washed with serum-free medium and incubated in Ham’s F-12 medium containing 1% Nutridoma-SP (Roche Diagnostics, Mannheim, Germany), 0.1% FBS, 10 µM sodium oleate-bovine serum albumin complex, and 10 µg/ml gentamicin for a given period [42]. HEK293 cells and SH-SY5Y cells were grown in Dulbecco’s modified Eagle’s medium (DMEM) supplemented with 10% (v/v) FBS in 5% CO_2_ at 37°C. ABCG1 expression was induced for 24 h by adding 5 µM TO901317 and 5 µM 9-cis retinoic acid to SH-SY5Y cells.

### Transfection of ABCG4

The expression plasmid pcDNA3.1Hygro(+)/ABCG4 was made by inserting human *ABCG4* cDNA [43] into the *Not*I and *EcoR*I sites of pcDNA3.1/Hygro(+) (Invitrogen). LY-A and LY-A/CERT cells were transfected with pcDNA3.1Hygro(+)/ABCG4 using LipofectAMINE (Invitrogen) according to the manufacturer’s instructions.

### Establishment of a stable transformant

The expression plasmid pcDNA3.1Hygro(+)/ABCA1 was made by introducing a termination codon into human ABCA1-mycHis [44]. The expression plasmid pcDNA3.1Hygro(+)/ABCG4-K120M was made by mutating the Walker A lysine to methionine by using the QuickChange II SiteDirected Mutagenesis Kit (Stratagene, La Jolla, CA). Next, HEK293 cells were transfected with pcDNA3.1Hygro(+)/ABCA1, pcDNA3.1Hygro(+)/ABCG4, or pcDNA3.1Hygro(+)/ABCG4-K120M using LipofectAMINE. Cells were selected with 350 µg/ml hygromycin for 10 days, and the expression of ABCA1 or ABCG4 was examined by Westernblotting.

### Cellular lipid release assay and methyl-β-cyclodextrin (MβCD) extraction

Cells were subcultured in 6-well plates at a density of 1.2×10^6^ cells. After incubation for the indicated period, the cells were washed with fresh medium and incubated with DMEM or Ham’s F-12 containing 0.02% bovine serum albumin (BSA) and 20 µg/ml HDL. The amounts of cholesterol and choline phospholipids in the medium were determined after 12 h of incubation as described previously [45]. Because the HDL added to the medium contained cholesterol and choline phospholipids, the efflux was calculated by subtracting the amounts of cholesterol and choline phospholipids in the HDL from those in the medium. To analyze the cholesterol available to cold MβCD extraction, cells were washed twice with cold PBS and incubated with DMEM containing 5 mM MβCD for 1 h on ice. The cholesterol content in the medium was determined as described previously [45].

### Treatment of cells with detergents

Cells were separated into detergent-soluble and -insoluble fractions as described previously [38]. Briefly, cells were washed with PBS and collected. The cells were suspended in MES buffer (25 mM MES (pH 6.5) and 150 mM NaCl) containing 1% Triton X-100 or 1% Brij 96, and incubated for 20 min on ice followed by centrifugation at 14,000×g for 20 min. The supernatant was pooled as a soluble fraction. The pellet was suspended in HEPES buffer (25 mM HEPES (pH 7.4) and 150 mM NaCl) containing 1% Triton X-100 or 1% Brij 96 and sonicated, then pooled as an insoluble fraction.

### OptiPrep gradient ultracentrifugation

Cells were washed with PBS and resuspended in TNE buffer (25 mM Tris-Cl (pH 7.4), 150 mM NaCl, and 5 mM EDTA containing 100 µg/ml (*p*-amidinophenyl)methanesulfonyl fluoride, 2 µg/ml leupeptin, and 2 µg/ml aprotinin) and passed through a 26G needle 10 times. Broken cells were centrifuged at 3,000×g for 5 min. TNE buffer containing 2% Triton X-100 or Brij 96 was added to ensure a final detergent concentration of 1%; then samples were incubated on ice for 15 min. Raft domains were isolated by a discontinuous OptiPrep gradient consisting of the following layers: 400 µl of 35% opti and lysates, 1,600 µl of 30% opti/TNE buffer, and 200 µl of TNE buffer [46]. The gradient was centrifuged in a TLS55 rotor (Beckman Coulter, Brea, CA) at 4°C for 4 h at 200,000×g. After centrifugation, ten 200-µl fractions were collected from the top of the tube and proteins were precipitated by acetone precipitation. The pellet was resuspended in a SDS-sample buffer and subjected to immunoblotting.

### Western blotting

Cells were washed with PBS and lysed in lysis buffer (50 mM Tris-Cl (pH 7.5), 150 mM NaCl, and 1% Triton X-100) containing protease inhibitors (100 µg/ml (*p*-amidinophenyl)methanesulfonyl fluoride, 2 µg/ml leupeptin, and 2 µg/ml aprotinin). Samples were electrophoresed on a 5–20% or 10% SDS-polyacrylamide gel and immunodetected with antibodies.

### Fluorescence microscopic analysis

HEK293 cells were transfected with pEGFP/flotillin-1-GFP plus pcDNA3.1(+)/ABCG1, pcDNA3.1(+)/ABCG1-K120M, pcDNA3.1(+)/ABCG4, or pcDNA3.1(+)/ABCG4-K120M. After 24 h of transfection, cells were subcultured on glass cover slips treated with poly-L-lysine (Sigma). After 48 h of transfection, cells were either fixed with cold methanol or with 4% paraformaldehyde in PBS+(phosphate-buffered saline containing 0.1 g/l of CaCl_2_ and MgCl_2_·6H_2_O), and permeabilized with 0.4% TritonX-100 in PBS+ for 5 min. To reduce nonspecific binding of antibodies, the cells were incubated in 10% goat serum in PBS+. Then, the cells were incubated for 1 h with a rabbit polyclonal anti-ABCG1 or anti-ABCG4 antibody, and then incubated with fluorescent Alexa546-conjugated anti-rabbit IgG (Invitrogen) for 1 h. Cells were directly visualized with a 63x Plan-Neofluar oil immersion objective using a Zeiss confocal microscope (LSM700).

### Cholera toxin binding to GM1

HEK293 cells were transfected with pcDNA3.1(−)/ABCG1-GFP, pcDNA3.1(−)/ABCG1-K120M-GFP, pcDNA3.1(−)/ABCG4-GFP, or pcDNA3.1(−)/ABCG4-K120M-GFP [47]. After 24 h of transfection, cells were subcultured on glass cover slips treated with poly-L-lysine (Sigma). After 48 h of transfection, cells were incubated with 5 µg/ml Alexa555-conjugated cholera toxin subunit B in PBS+ for 5 min on ice. Cells were fixed with 4% paraformaldehyde and viewed using a confocal microscope.

### Statistical analysis

Values are presented as means ± S.D. Multiple comparisons were performed using ANOVA with the Dunnet test. A value of *P*<0.05 was considered statistically significant.

## Results

### Cholesterol efflux mediated by ABCG4 is not affected by reduced cellular SM levels

Although ABCG1, ABCG4, and ABCA1 mediate the efflux of cholesterol when they are expressed in HEK293 cells, they differ in their abilities to efflux phospholipids. We previously showed that ABCG1 mediates the efflux of choline phospholipids (mainly SM) to HDL, whereas ABCA1 mediates the efflux of choline phospholipids (mainly PC) to lipid-free apoA-I in HEK293 cells [10]. In accordance with the previous study, HEK293 cells stably expressing ABCA1 and ABCG1 (HEK/ABCA1 and HEK/ABCG1, respectively) mediated the efflux of both cholesterol and choline phospholipids, as shown by lipid mass release (Fig. S1) and fractional lipid release (Fig. S2). As reported by Wang *et al.* [14], HEK/ABCG4 cells mediated the efflux of cholesterol to HDL, but did not mediate the efflux of choline phospholipids, including PC and SM (Figs. S1 and S2) (Table 1). Neither cholesterol nor choline phospholipids were exported from HEK/ABCG4-KM cells, which stably expressed a non-functional ABCG4 (a Walker A lysine mutant), indicating that cholesterol export by ABCG4 is ATPase dependent.

**Table 1:** Comparison of lipid efflux mediated by ABCA1, ABCG1, and ABCG4.

	ABCA1	ABCG1	ABCG4
Substrates	C, PC	C, SM	C
Impact of decreasingSM levels	↑	↓	→
Impact of increasingSM levels	↓	↑	n.d.

C, cholesterol; PC, phosphatidylcholine; SM, sphingomyelin; n.d., not determined.

↑, ↓, or →, Lipid efflux increased, decreased, or did not change, respectively.

**Figure 1:**
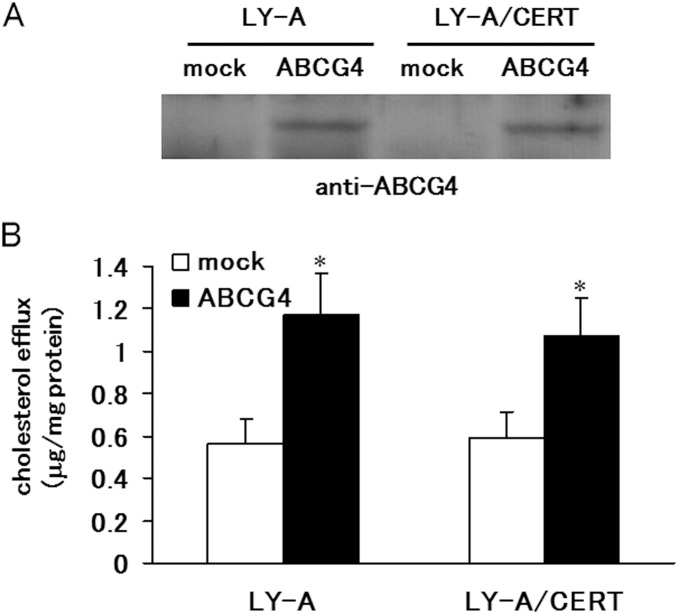
Efflux of cholesterol from LY-A and LY-A/CERT cells. LY-A cells or LY-A/CERT cells, preincubated in Nutridoma-BO medium for 40 h, were mock-transfected (open bars) or transfected with ABCG4 (filled bars). After 18 h of the transfection, cell lysates (10 µg) were separated by 10% polyacrylamide gel electrophoresis, and ABCG4 was detected with immunoblotting (A). After 6 h of the transfection, the efflux of cholesterol from cells during 12 h in the presence of 0.02% BSA plus 20 µg/ml HDL was analyzed (B). Average values of three experiments are presented with the SD. *P<0.05, significantly different from mock-transfected cells.

When the cellular SM levels are reduced, lipid efflux mediated by ABCG1 was impaired [48], whereas lipid efflux mediated by ABCA1 was stimulated [39]. To examine the effects of cellular SM levels on the cholesterol efflux by ABCG4, we expressed human ABCG4 in the mutant CHO-K1 cell line, LY-A, which has a mutation in the ceramide transfer protein, CERT. The expression levels of ABCG4 were similar between LY-A cells and the control LY-A/CERT cells; in the latter cell type, ceramide trafficking function is recovered by the stable expression of human CERT (Fig. 1A). The expression of ABCG4 increased cholesterol levels in the medium in both LY-A and LY-A/CERT cells as compared to mock-transfected cells (Fig. 1B). The amounts of cholesterol exported by ABCG4 from LY-A and LY-A/CERT cells did not differ significantly (Fig. 1B), although the SM levels in LY-A cells have been found to be reduced by about 36% compared with that in LY-A/CERT cells [48]. This observation indicates that cellular SM levels do not affect cholesterol efflux by ABCG4, in contrast to ABCA1 and ABCG1 (Table 1).

### ABCA1, ABCG1, and ABCG4 are distributed to distinct meso-domains on the plasma membrane

**Figure 2:**
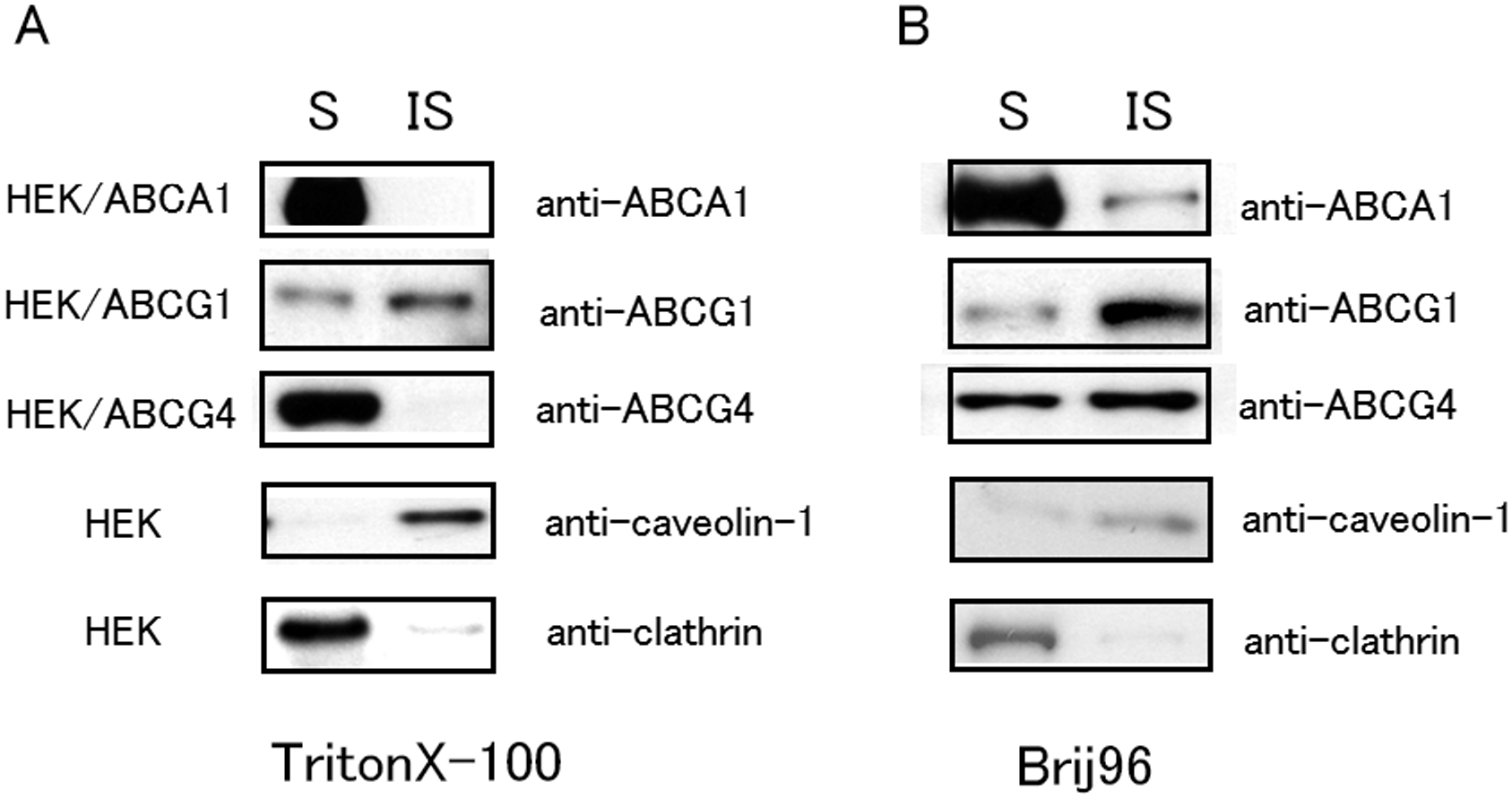
Solubility of ABCA1, ABCG1, and ABCG4 treated with Triton X-100 and Brij 96. HEK293, HEK/ABCA1, HEK/ABCG1, or HEK/ABCG4 cells were treated with 1% Triton X-100 (A) or Brij 96 (B), and separated into soluble (S) and insoluble (IS) fractions by centrifugation. The soluble and insoluble fractions (5 µg of proteins) were separated by 10% polyacrylamide gel electrophoresis, and ABCA1, ABCG1, ABCG4, caveolin-1, or clathrin HC were detected by immunoblotting.

Because SM contributes to the formation of raft domains, with which cholesterol and SM are associated, we speculated that the differential efflux of ABCA1, ABCG1, and ABCG4 in response to cellular SM levels might be caused by the differential distribution of ABC proteins at the plasma membrane. To examine this possibility, HEK/ABCA1, HEK/ABCG1, and HEK/ABCG4 cells were solubilized by Triton X-100 and Brij 96 (Fig. 2). When HEK293 cells were treated with Triton X-100, caveolin-1, a raft marker protein, was recovered from an insoluble fraction, whereas clathrin heavy chain, a non-raft marker protein, was recovered from a soluble fraction (Fig. 2A). As reported previously [38], [39], ABCA1 was localized to non-raft domains; ABCA1 was recovered from a soluble fraction when it was treated with Triton X-100 (Fig. 2A and Fig. S3). Similarly, most of ABCA1 was detected in the soluble fraction when cells were treated with Brij 96 (Fig. 2B and Fig. S3). In contrast to ABCA1, ABCG1 was preferentially recovered from an insoluble fraction when HEK/ABCG1 cells were treated with Triton X-100 or Brij 96. This suggests that ABCG1 is localized to raft domains in the plasma membrane. ABCG4 was recovered from the soluble fraction when treated with Triton X-100, but equally recovered from soluble and insoluble fractions when treated with Brij 96. These results suggest that ABCA1, ABCG1, and ABCG4 are distributed to distinct meso-domains on the plasma membrane: ABCA1 is localized to non-raft domains, ABCG1 to Triton X-100 raft domains, and ABCG4 to Brij 96 raft but not to Triton X-100 raft domains.

### ABCG1 and ABCG4 are localized to raft domains, while ABCA1 is localized to non-raft domains

**Figure 3:**
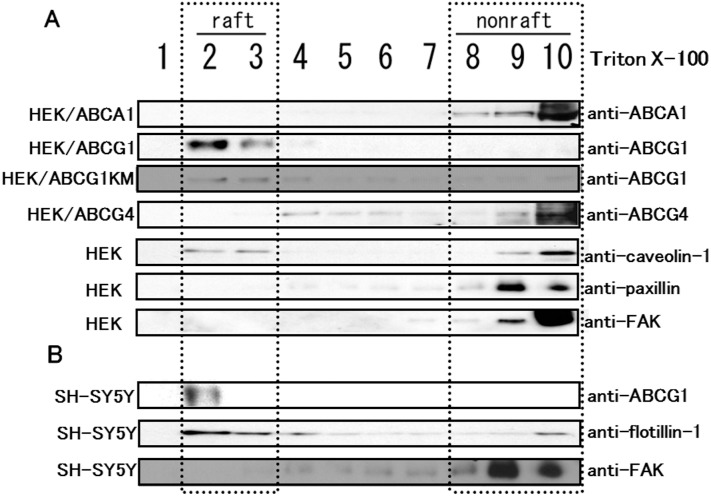
Distribution of ABCA1, ABCG1, and ABCG4 between Triton X-100 raft and non-raft domains. HEK293, HEK/ABCA1, HEK/ABCG1, HEK/ABCG1-KM, or HEK/ABCG4 cells (A) or SH-SY5Y cells (B) treated with TO901317 and 9-cis retinoic acid for 24 h were incubated with buffer containing 1% Triton X-100 on ice. The cell lysates were separated by OptiPrep-gradient ultracentrifugation. Ten fractions of each were separated by 5–20% polyacrylamide gel electrophoresis, and ABCA1, ABCG1, ABCG4, caveolin-1, paxillin, FAK, or flotillin was detected by immunoblotting.

To exclude the possibility that ABCG1 and ABCG4 were recovered from the insoluble fraction due to interactions with the cytoskeleton and to confirm that ABCG1 and ABCG4 were localized to raft domains, proteins were separated by OptiPrep gradient ultracentrifugation after treatment with Triton X-100 (Fig. 3A). When endogenous caveolin-1 (a raft marker), paxillin (a non-raft marker), and FAK (a non-raft marker) expressed in HEK293 cells were separated, caveolin-1 was found in fractions 2 and 3 (raft fractions), and both paxillin and FAK were detected only in fractions 8–10 (non-raft fractions). Under these conditions, the distribution of ABCA1, ABCG1, and ABCG4 expressed in HEK293 cells was examined. ABCA1 was detected in fractions 8–10, suggesting that ABCA1 is localized to non-raft domains. By contrast, ABCG1 was exclusively detected in fractions 2 and 3, suggesting that ABCG1 is localized to raft domains. ABCG1-KM, a non-functional ABCG1 (a Walker A lysine mutant), was also found in fractions 2 and 3 like wild-type ABCG1, suggesting that ATP hydrolysis is not required for the localization of ABCG1 to raft domains. ABCG4 was detected both in fractions 4–6 and fractions 9–10, but not in fractions 2 and 3. These results suggest that ABCG4 is not localized to Triton X-100 raft domains, and that the distribution of ABCG4 is different from that of ABCA1 or ABCG1. Next, we examined the distribution of endogenously expressed ABCG1 in neuroblastoma SH-SY5Y cells (Fig. 3B). When SH-SY5Y cells were treated with Triton X-100 followed by gradient ultracentrifugation, flotillin (a raft marker) was detected in fractions 2 and 3, and was faintly detected in fractions 4–10. FAK was predominantely detected in fractions 8–10. ABCG1 was exclusively detected in fraction 2. These results indicate that ABCG1 is localized to Triton X-100 raft domains, in contrast to ABCA1 and ABCG4. Furthermore, localizations of ABCA1 and ABCG4 also differ.

**Figure 4:**
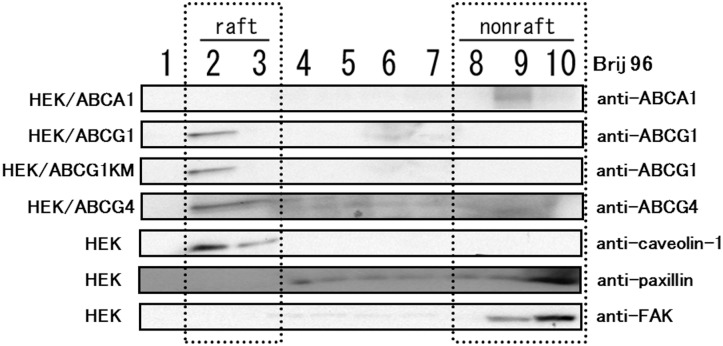
Distribution of ABCA1, ABCG1, and ABCG4 between Brij 96 raft and non-raft domains. HEK293, HEK/ABCA1, HEK/ABCG1, HEK/ABCG1-KM, or HEK/ABCG4 cells were incubated with buffer containing 1% Brij 96 on ice. The cell lysates were separated by OptiPrep-gradient ultracentrifugation. Ten fractions of each were separated by 5–20% polyacrylamide gel electrophoresis, and ABCA1, ABCG1, ABCG4, caveolin-1, paxillin, or FAK were detected by immunoblotting.

To confirm that ABCG4 is localized to Brij 96 raft domains, proteins were separated by OptiPrep gradient ultracentrifugation after treatment with Brij 96 (Fig. 4). Endogenous caveolin-1 was detected in fractions 2 and 3, while paxillin and FAK were mainly detected in fractions 8–10. ABCA1 was found in fraction 9, suggesting that ABCA1 is localized to non-raft domains. ABCG1 and ABCG1-KM were detected exclusively in fraction 2, and ABCG4 was detected in fractions 2 and 3. These results indicate that ABCG1 is localized to domains resistant to both Triton X-100 and Brij 96 (Triton X-100 raft) and that ABCG4 is localized to domains resistant to Brij 96 but not to Triton X-100 (Brij 96 raft), whereas ABCA1 is localized to non-raft domains.

### ABCG1 is colocalized with flotillin-1

**Figure 5:**
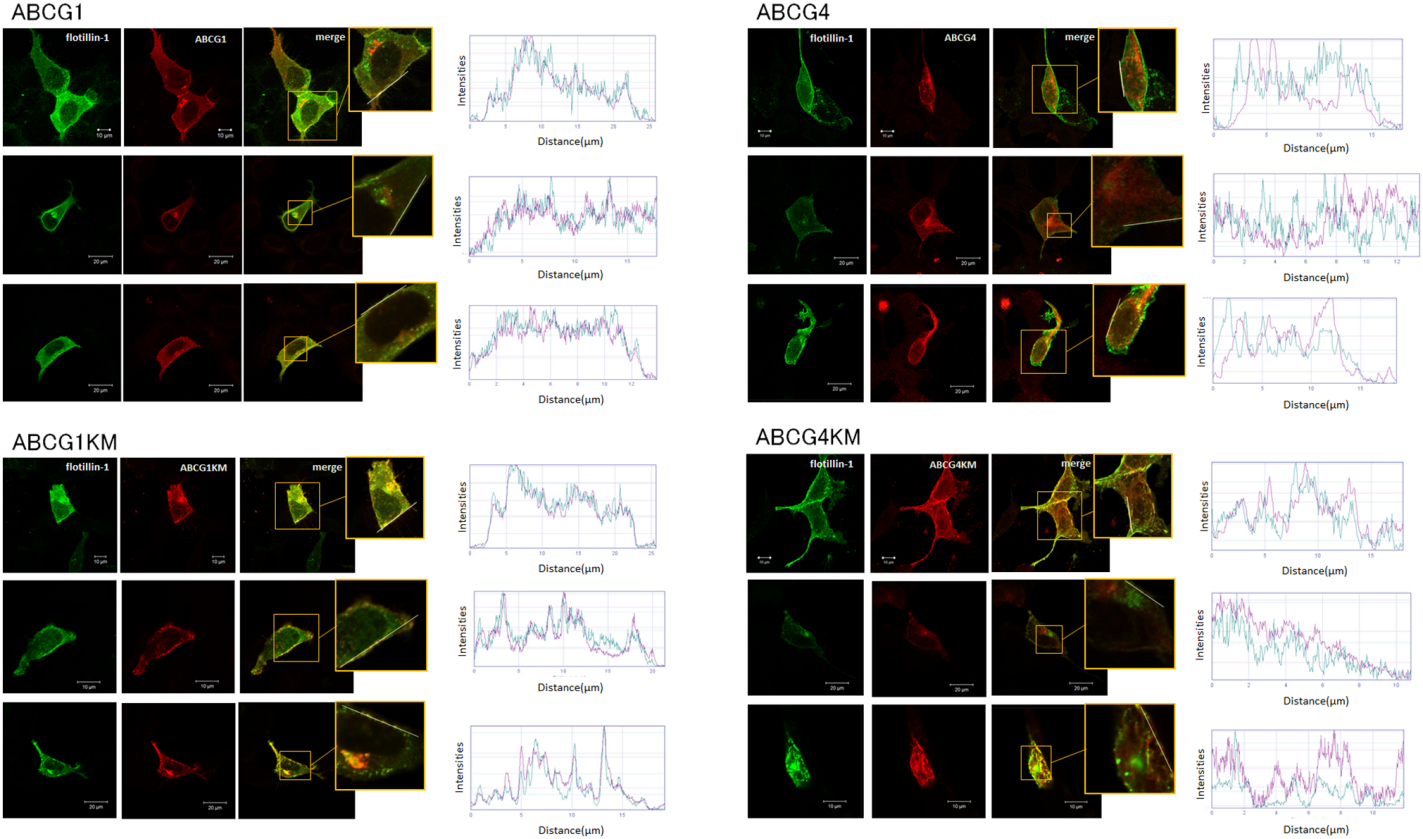
Colocalization of ABCG1 with flotillin-1. HEK293 cells transiently expressing ABCG1, ABCG1-KM, ABCG4, or ABCG4-KM plus flotillin-GFP were permeabilized with Triton X-100 (ABCG1) or methanol (ABCG4) and reacted with anti-ABCG1 or -ABCG4 antibodies and analyzed by confocal microscopy. Line scans analyzed by ImageJ software indicate the fluorescence intensities of flotillin-1 (green), ABCG1 (red), and ABCG4 (red).

Biochemical analysis suggests that ABCG1 and ABCG4 localized to the raft domains. To confirm this, we examined the subcellular localization of ABCG1 and ABCG4 on the plasma membrane by investigating the colocalization of ABCG1 and ABCG4 with raft marker proteins. ABCG1, ABCG1-KM, ABCG4, or ABCG4-KM fused with GFP was expressed in HEK293 cells and endogenous caveolin-1 was detected with an anti-caveolin-1 antibody. Neither ABCG1 nor ABCG4 was colocalized with caveolin-1 as reported previously [40], though they were localized to the plasma membrane (data not shown). Next, we investigated the colocalization of ABCG1 and ABCG4 with flotillin-1. Because an antibody that recognizes endogenous flotillin in HEK293 cells was not available, ABCG1 or ABCG4 was coexpressed with GFP-fused flotillin-1. When ABCG1 or ABCG4 was immunostained with the antibody, ABCG1 and ABCG1-KM were colocalized with flotillin-1 (Fig. 5). However, ABCG4 and ABCG4-KM were only partially colocalized with flotillin-1. These results suggest that ABCG1 localized to raft domains containing flotillin-1, and that ABCG1 and ABCG4 localized to distinct membrane domains on the plasma membrane.

### ABCG1 and ABCG4 increase MβCD-extractable cholesterol

**Figure 6:**
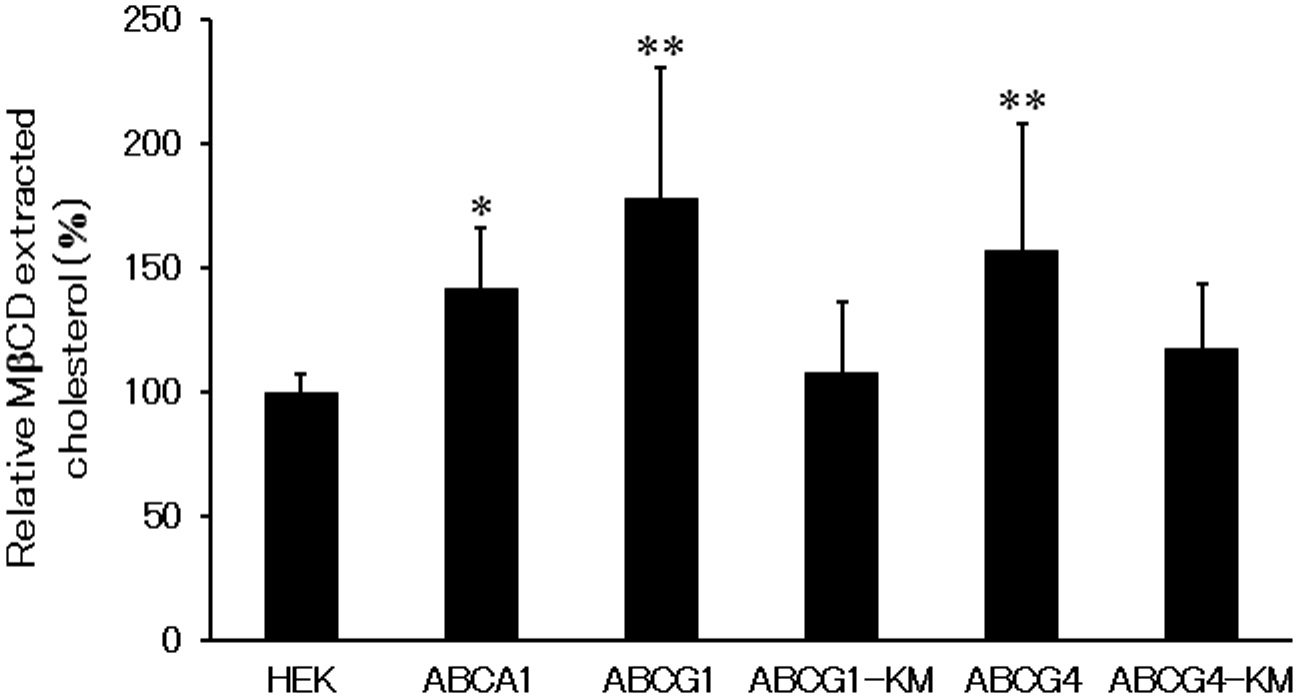
Cholesterol extraction by cold MβCD. HEK293, HEK/ABCA1, HEK/ABCG1, HEK/ABCG1-KM, HEK/ABCG4, or HEK/ABCG4-KM cells were incubated with the medium containing 5 mM MβCD on ice for 1 h. The amount of cholesterol extracted by MβCD from each cell type is presented relative to the amount extracted from HEK293 cells. Average values of 3–9 experiments are presented with the SD. *P<0.05; **P<0.01, significantly different from HEK293 cells.

We speculated that ABCG1 and ABCG4 disrupt raft domains and increase the size of non-raft domains by reorganizing lipids in raft domains. To see if ABCG1 and ABCG4 augment non-raft domains, we investigated the amounts of cholesterol available to extraction by cold MβCD. When cells were incubated with MβCD on ice, MβCD extracted 41% more cholesterol from HEK/ABCA1 cells than from host HEK293 cells (Fig. 6). HEK/ABCG1 and HEK/ABCG4 cells also showed significant increases of cholesterol (78% and 57% increases, respectively, as compared to HEK293 cells) extracted by cold MβCD, but neither HEK/ABCG1-KM nor HEK/ABCG4-KM cells did. An increase of MβCD-extracted cholesterol was also observed in baby hamster kidney (BHK) cells in which ABCG1 expression had been induced (data not shown). These results show that ABCG1 and ABCG4 reorganize raft domains in an ATPase-dependent manner and increase the cholesterol content in non-raft domains.

### ABCG1 and ABCG4 disturb the distribution of caveolin-1 to raft domains

**Figure 7:**
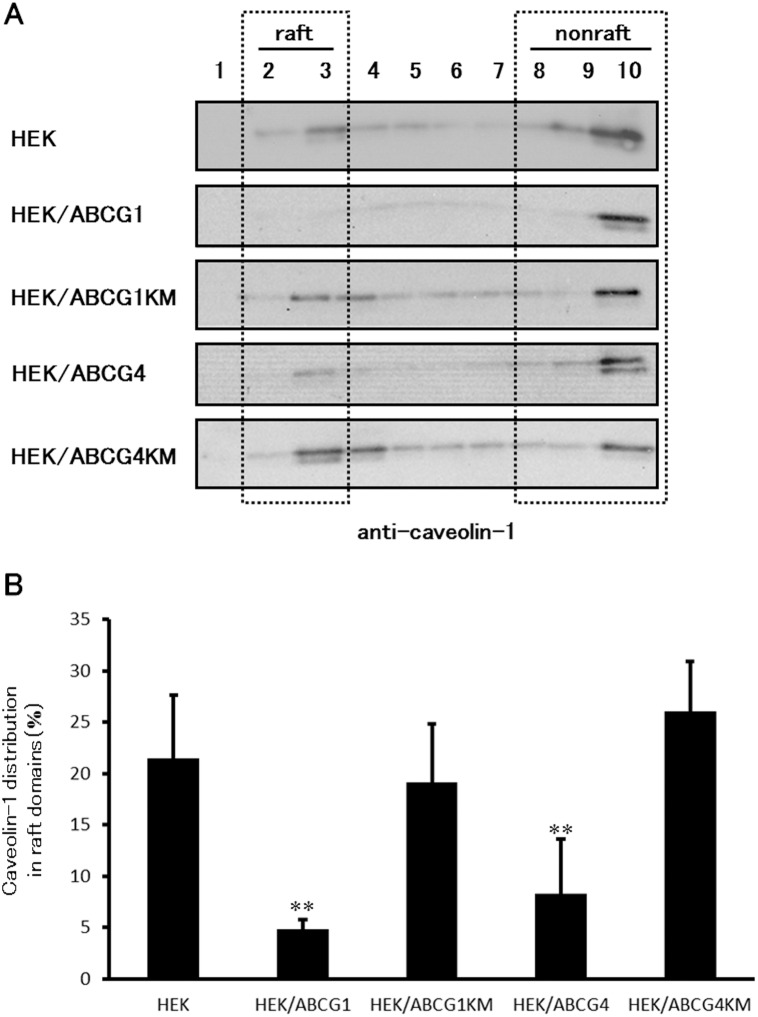
Distribution of caveolin-1 between Triton X-100 raft and non-raft domains. (A) HEK293, HEK/ABCG1, HEK/ABCG1-KM, HEK/ABCG4, or HEK/ABCG4-KM cells were cultured in DMEM containing 0.2% BSA for 20 h and incubated with buffer containing 1% Triton X-100 on ice. The cell lysates were separated by OptiPrep-gradient ultracentrifugation. Ten fractions of each were separated by 5–20% polyacrylamide gel electrophoresis, and caveolin-1 was detected by immunoblotting. (B) The amount of caveolin-1 detected by immunoblotting was analyzed. The data represent the percentage of caveolin-1 in the raft domains (fractions 2 and 3) relative to the total caveolin-1 (fractions 1–10). Average values of 3–7 experiments are presented with the SD. **P<0.01, significantly different from HEK293 cells.

To verify that ABCG1 and ABCG4 reorganize raft domains, we examined the distribution of caveolin-1 on the plasma membrane (Fig. 7). When HEK293 cells were treated with 1% Triton X-100 followed by OptiPrep gradient ultracentrifugation (Fig. 7A), caveolin-1 from HEK293 cells was detected in fractions 2 and 3 like in Fig. 3A. Caveolin-1 was detected in raft fractions in HEK/ABCG1-KM and HEK/ABCG4-KM cells, but not in HEK/ABCG1 cells, and was hardly seen in HEK/ABCG4 cells. About 20% of caveolin-1 was distributed to raft domains in HEK293 cells, but only 5 and 8% of caveolin-1 was detected in raft domains of HEK/ABCG1 and HEK/ABCG4 cells, respectively (Fig. 7B), suggesting that ABCG1 and ABCG4 reorganize membranes and disrupt raft domains. The distribution of caveolin-1 to raft domains in HEK/ABCG1-KM and HEK/ABCG4-KM cells was not significantly different from that in HEK293 cells, indicating that the disruption of raft domains is ATPase-dependent. The result was also confirmed in BHK cells in which ABCG1 exression had been induced (data not shown). These experiments were carried out after cells had been incubated in medium without HDL or serum but containing BSA. Therefore, lipid reorganization but not lipid efflux is involved in the disruption of raft domains by ABCG1 or ABCG4.

### ABCG1 and ABCG4 disturb cholera toxin binding to GM1

**Figure 8:**
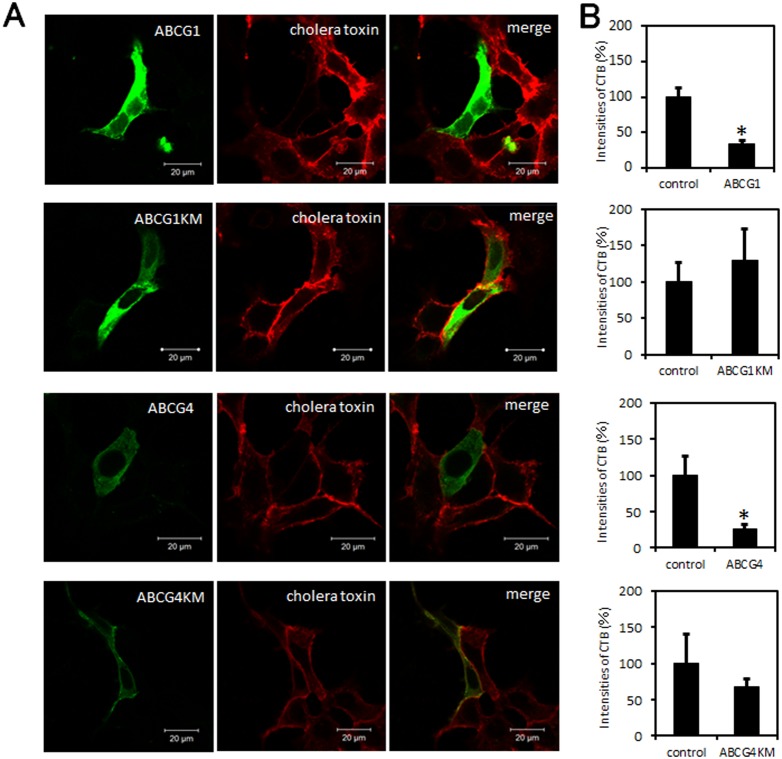
Cholera toxin binding to GM1. (A) HEK293 cells transiently expressing ABCG1-GFP, ABCG1-KM-GFP, ABCG4-GFP, or ABCG4-KM-GFP were incubated with Alexa555-conjugated cholera toxin on ice and fixed with 4% paraformaldehyde. (B) The intensities of cholera toxin (CTB) fluorescence per cell expressing ABCG1, ABCG1-KM, ABCG4, or ABCG4-KM were calculated using ImageJ software and are showed relative to the signal obtained from a control cell that did not express ABCG proteins. *P<0.05, significantly different from control cells.

An ultracentrifugation assay suggested that ABCG1 and ABCG4 affect raft domains. To examine if the disruption of raft domains occurs in cells, the distribution of GM1 on the plasma membrane was analyzed using confocal microscopy. Cholera toxin specifically binds to GM1, which accumulates in raft domains. When cells transiently expressing ABCG1-GFP or ABCG4-GFP were treated with fluorescence-labelled cholera toxin on ice to avoid endocytosis, the intensity of immunofluorescence was significantly less as compared to that observed in cells that did not express ABCG1 or ABCG4 (Fig. 8). By contrast, expression of ABCG1-KM or ABCG4-KM did not change the fluorescence intensity of staining relative to the control. These results suggest that ABCG1 and ABCG4 affect the raft domain structure and disturb the distribution of GM1 in an ATPase activity-dependent manner.

## Discussion

**Figure 9:**
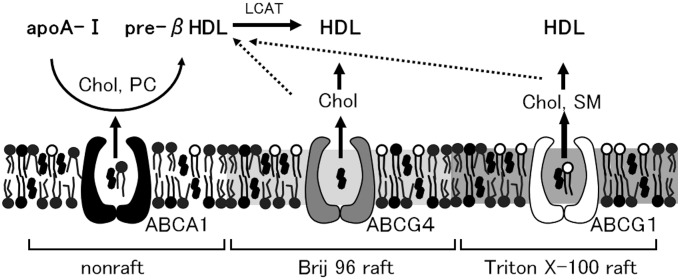
Localization and lipid efflux of ABCA1, ABCG1, and ABCG4 on the plasma membrane. ABCA1 is localized to non-raft domains, where ABCA1 mediates the efflux of cholesterol (chol) and phosphatidylcholine (PC) to apoA-I and functions in the formation of preβ-HDL. The cholesterol in the preβ-HDL is esterified by lecithin-cholesterol acyl transferase (LCAT), which forms HDL. ABCG4 is localized to Brij 96 raft domains, where ABCG4 mediates the efflux of cholesterol to HDL. ABCG1 is localized to Triton X-100 raft domains, where ABCG1 mediates the efflux of cholesterol and sphingomyelin (SM) to HDL.

In this study, we examined the distributions of ABCA1, ABCG1, and ABCG4 on the plasma membrane and demonstrated that ABCA1, ABCG1, and ABCG4 are distributed to distinct membrane meso-domains (Fig. 9); ABCA1 is localized to non-raft domains, whereas ABCG1 and ABCG4 are localized to Triton X-100 raft domains and Brij 96 raft domains, respectively. Furthermore, ABCG1, but not ABCG4, is colocalized with flotillin on the plasma membrane. This is the first report to show the differential localization of ABCG1 and ABCG4 to membrane meso-domains. Furthermore, we showed that ABCA1, ABCG1, and ABCG4 disturb lipid raft domains.

The distinct localizations of ABCA1, ABCG1, and ABCG4 may determine their differential transport substrates (Fig. 9). Although ABCA1 seems to recognize both PC and SM [49], it preferentially mediates the efflux of PC [10]. This may be because ABCA1 is localized to non-raft domains where PC is rich. ABCG1 mediates the efflux of both PC and SM, but preferentially the efflux of SM [10]. Triton X-100 raft domains, where ABCG1 is localized, are rich in cholesterol and SM [8]. Thus, ABCG1 may recognize SM as a transport substrate rather than PC. This likelihood is supported by our previous study that purified ABCG1 seems to interact with cholesterol and choline phospholipids, especially SM [47]. ABCG4 is localized to Brij 96 raft domains enriched in cholesterol [8], and mediates the efflux of cholesterol but not choline phospholipids, as shown in this study and previously [14]. In the membrane meso-domains where ABCG4 functions, choline phospholipids may not be available for transport. Alternatively, ABCG4 may have differential mechanisms of substrate recognition and transport compared with ABCA1 and ABCG1. Preβ-HDL formed by ABCA1 contains relatively more PC and less SM compared with HDL formed by ABCG1 [10]. The SM-poor preβ-HDL could be a good acceptor of cholesterol and SM transported by ABCG1. ABCG4, expressed in the central nervous system, would mediate the efflux of cholesterol to the PC-rich apoE-HDL formed by ABCA1. The different substrates of ABCA1, ABCG1, and ABCG4 may be important for the production of HDL composed of various lipids. Furthermore, the contents of cholesterol, PC, and SM in HDL may be regulated by the balanced efflux mediated by ABCA1, ABCG1, and ABCG4.

We have shown that lipid effluxes mediated by ABCA1 and ABCG1 are increased and impaired, respectively, by a decrease of cellular SM levels [39], [48]. In this study, we showed that the efflux of cholesterol mediated by ABCG4 is not affected by decreased SM levels. The distinct localizations of ABCA1, ABCG1, and ABCG4 on the plasma membrane may account for the different effects of cellular SM levels on their functions. A membrane environment of lipid rafts that are thick and liquid-ordered might be required for the function of ABCG1. Decreased SM levels disrupt raft domains, where ABCG1 resides, leading to the decreased activity of ABCG1. On the other hand, a decrease of SM level would be favorable for ABCA1 because it enlarges the area where ABCA1 functions. ABCG4 is localized to Brij 96 raft domains and partly to non-raft domains as shown in Fig. 2. This unsettled localization of ABCG4 may explain the observation that decreased SM levels had no effect on its activity. It has been reported that cellular SM content is increased in macrophages treated with acetyl-low density lipoprotein (LDL) [50], in macrophages during differentiation from monocyte [51], in peritoneal macrophages during aging of rats [52], and in alveolar macrophages during postnatal development [53]. Changes in cellular SM levels may be involved in regulating the function of ABCA1 and ABCG1, and in the development of atherosclerosis.

Mendez *et al.* have reported that apoA-I removes cellular cholesterol from Triton X-100-soluble membranes, and that HDL removes cholesterol from Triton X-100-soluble and -insoluble membranes [38]. These findings are in accord with our result that ABCA1 and ABCG1 were localized to Triton X-100-soluble and -insoluble domains, respectively. We suggest that apoA-I removes cholesterol from non-raft domains, where ABCA1 resides, and that HDL removes cholesterol both from raft domains, where ABCG1 resides, and from non-raft domains, possibly by simple diffusion. Because cholesterol, newly synthesized or derived from lipoproteins like LDL, is trafficked to raft domains [54], ABCG1 and ABCG4 may function in the “on-demand” removal of cholesterol from raft domains, when cellular cholesterol levels of macrophages becomes high through the endocytosis of LDL or engulfment. By contrast, ABCA1 may function in the “housekeeping” removal of cholesterol from non-raft domains, because detectable amounts of ABCA1 proteins are expressed in macrophages, fibroblasts, and astrocytes, even when intracellular cholesterol levels are not high [39], [55], [56]. Furthermore, ABCG1 mediates the efflux of 7-ketocholesterol [15], which is incorporated into raft domains and induces cell death [57]. When 7-ketocholesterol is associated with raft domains, ABCG1 may remove 7-ketocholesterol rapidly from raft domains in order to protect cells from the toxicity of 7-ketocholesterol. The physiological significance of the distinct distribution of ABCA1, ABCG1, and ABCG4 in the plasma membrane may be related to the different roles among these ABC proteins on the sterol efflux *in vivo*.

Although ABCA1, ABCG1, and ABCG4 are localized to distinct membrane meso-domains, they all seem to disturb raft domain structures, as shown in Fig. 6, 7, and 8. It has been shown that ABCA1 and ABCG1 increase the amounts of cholesterol accessible to cholesterol oxidase [32], [33], and that ABCA1 increases the amount of cholesterol available to cold MβCD extraction [31], [39]. Similarly, we showed that ABCA1, ABCG1, and ABCG4 increased the amount of cholesterol extracted by cold MβCD in Fig. 6, suggesting that ABCA1, ABCG1, and ABCG4 increase the area of non-raft domains. ABCG1 and ABCG4 decreased the distribution of caveolin-1 to raft domains in our study, and ABCA1 has also been reported to alter the distribution of caveolin-1 [31], suggesting that ABCA1, ABCG1, and ABCG4 disturb raft domains. Furthermore, ABCG1 and ABCG4 decreased cholera toxin binding to GM1 as shown in Fig. 8. This is coincident with a study showing cholera toxin binding was increased in macrophages from Abcg1 knockout mice [58]. Together, these findings suggest that ABCA1, ABCG1, and ABCG4 disrupt raft domains. The mechanism of the disruption of the raft domains remains elusive, but we propose that ABCA1, ABCG1, and ABCG4 transport lipids in the plasma membrane, thereby reducing the interactions of these lipids with other lipids, with proteins including caveolin-1, and/or with gangliosides including GM1, leading to the reorganization of lipids and the disruption of raft domains. The fact that the three cholesterol transporters mediate similar effects on raft domains suggests that cholesterol efflux by ABCA1, ABCG1, and ABCG4 is based on similar mechanisms. The molecular mechanisms underlying cholesterol efflux to apoA-I or HDL may be that ABCA1, ABCG1, and ABCG4 provide easily removable cholesterol, which is extracted by apoA-I or HDL, by reorganizing membrane meso-domains. However, we cannot exclude the possibility that other mechanisms also affect cholesterol efflux.

It has been reported that several ABC proteins are localized to raft domains. Ismair *et al.* showed that Abcb11 (Bsep), Abcb4 (Mdr2), Abcc2 (Mrp2), and Abcg5 are localized to Lubrol raft domains of the canalicular membrane in hepatocytes [59]. ABCB1 (MDR1) resides in Triton X-100 raft domains [59], [60] or Brij 96 raft domains [61]. Cholesterol depletion inhibited drug transport by ABCB1 [62]. ABCG2 was also localized to Triton X-100 raft domains [63]. It is not clear how ABCG1 and ABCG4 are localized to the raft domains. ABCG1 and ABCG4 may interact with raft resident proteins because ABCG2 has been reported to interact with caveolin [63]. Alternatively, properties of transmembrane segments of ABCG1 and ABCG4, such as the length of the transmembrane helices and/or the interactions of transmembrane helices with cholesterol and SM, favor the localization of the proteins in raft domains, because raft domains are rich in cholesterol and SM and have a thicker lipid bilayer than non-raft domains.

In the plasma membrane, various meso-scale (10–100 nm) domains, such as lipid rafts, are supposed to be dynamically organized and re-organized and involved in various cellular functions, such as signal transduction and endocytosis [9]. Thus, ABCA1, ABCG1, and ABCG4 could influence many physiological phenomena by disturbing raft structures and modulating reactions that occur in raft domains. Indeed, expression of ABCA1 or ABCG1 downregulated Akt phosphorylation by reducing lipid raft size [22], [31]. Furthermore, the expression of ABCG1 or ABCG4 reduced production of amyloidβ by disturbing the localization of γ-secretase to raft domains (manuscript in preparation). ABCA1 and ABCG1 suppress inflammatory responses of macrophages [58], [64], [65], [66], [67]. ABCG1 is involved in the regulation of T-cell proliferation [19] and apoptosis of macrophages [20], and promotes endothelial NO synthesis by decreasing the interaction of caveolin-1 and NO synthase [40]. ABCG4 suppresses platelet production by regulating megakaryocyte progenitors proliferation [23]. The disturbance of raft structures by ABCA1, ABCG1, and ABCG4 might be involved in these phenomena.

In summary, we have demonstrated that ABCA1, ABCG1, and ABCG4 are different in their localizations on the plasma membrane, in their transport substrates, and in the effect of cellular SM levels on their lipid transport activities. We have also shown that ABCA1, ABCG1, and ABCG4 disturb the distribution of caveolin-1 to the raft domains independently of lipid acceptors. These results suggest that the distinct localizations of ABCA1, ABCG1, and ABCG4 on the plasma membrane may determine their different transport substrates and responses to cellular SM levels. Furthermore, we propose that ABCA1, ABCG1, and ABCG4 reorganize membrane meso-domains, leading to the disturbed raft domains. The disruption of raft domains may enhance the efflux of cholesterol from the plasma membrane, and may affect reactions occurring in raft domains such as signal transduction in the immune response. This study would facilitate our understanding of the mechanism and physiological roles of lipid efflux mediated by ABCA1, ABCG1, and ABCG4.

## Supporting Information

Figure S1 **Efflux of cellular cholesterol and phospholipids by ABCG1 or ABCG4.** The efflux of cholesterol (A) and phospholipids (B) from HEK293, HEK/ABCG4, HEK/ABCG4-KM, or HEK/ABCG1 cells during 24 h in the presence of 0.02% BSA alone (white bars) or 0.02% BSA plus 20 µg/ml HDL was analyzed. Average values of three experiments are presented with the SD. **P<0.01, significantly different from HEK293 cells.
(TIF)

Figure S2 **Efflux of fractional [^3^H]cholesterol and [^3^H]choline phospholipids by ABCA1 or ABCG4.** Cells were labeled for 24 h with [^3^H]cholesterol or [^3^H]choline in DMEM containing 10% FBS, and the efflux of [^3^H]cholesterol (A) or [^3^H]choline phospholipids (B) from HEK293, HEK/ABCG4, HEK/ABCG4-KM, or HEK/ABCA1 cells during 4 h in the presence of 0.02% BSA alone (white bars), 0.02% BSA plus 10 µg/ml apoA-I (light gray bars), 0.02% BSA plus 10 µg/ml apoE (dark gray bars), or 0.02% BSA plus 20 µg/ml HDL (black bars) was analyzed. Average values of three experiments are presented with the SD. *P<0.05; **P<0.01, significantly different from HEK293 cells.
(TIF)

Figure S3 **Solubility of ABCA1, ABCG1, and ABCG4 treated with Triton X-100 and Brij 96.** The raw data for Fig. 2 are shown. HEK293, HEK/ABCA1, HEK/ABCG1 (clone #A62), HEK/ABCG1 (clone #B9), or HEK/ABCG4 cells were treated with 1% Triton X-100 or Brij 96, and separated to soluble (S) and insoluble (IS) fractions by centrifugation.
(TIF)
