# Supplementary figures and images for: ABCA1, ABCG1, and ABCG4 Are Distributed to Distinct Membrane Meso-Domains and Disturb Detergent-Resistant Domains on the Plasma Membrane
Source: PLoS One. 2014 Oct 10;9(10):e109886. doi: 10.1371/journal.pone.0109886 (PMC4193829; doi:10.1371/journal.pone.0109886)

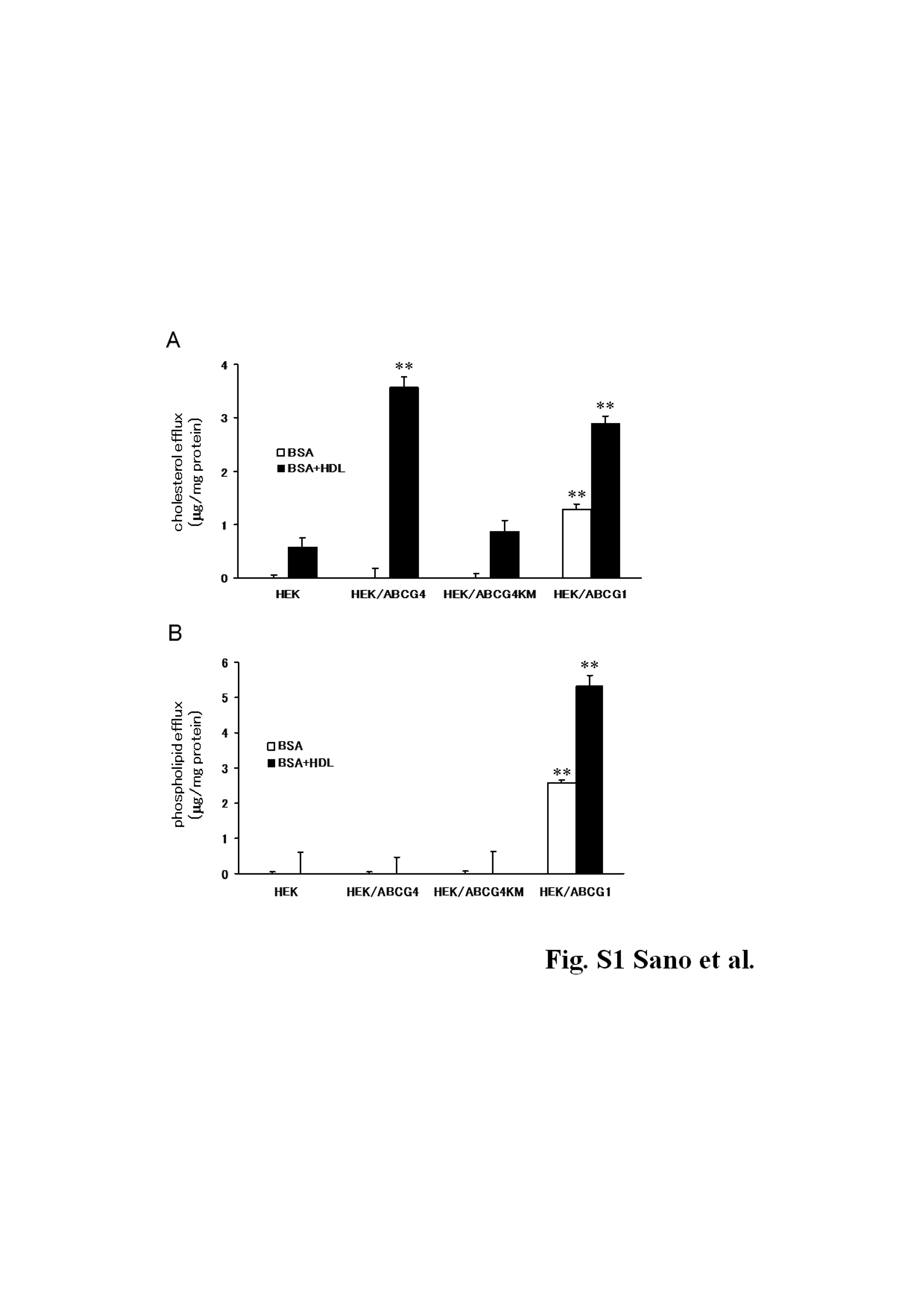

Supplement: Figure S1 — Efflux of cellular cholesterol and phospholipids by ABCG1 or ABCG4. The efflux of cholesterol (A) and phospholipids (B) from HEK293, HEK/ABCG4, HEK/ABCG4-KM, or HEK/ABCG1 cells during 24 h in the presence of 0.02% BSA alone (white bars) or 0.02% BSA plus 20 µg/ml HDL was analyzed. Average values of three experiments are presented with the SD. **P<0.01, significantly different from HEK293 cells. (TIF) [file pone.0109886.s001.tif]

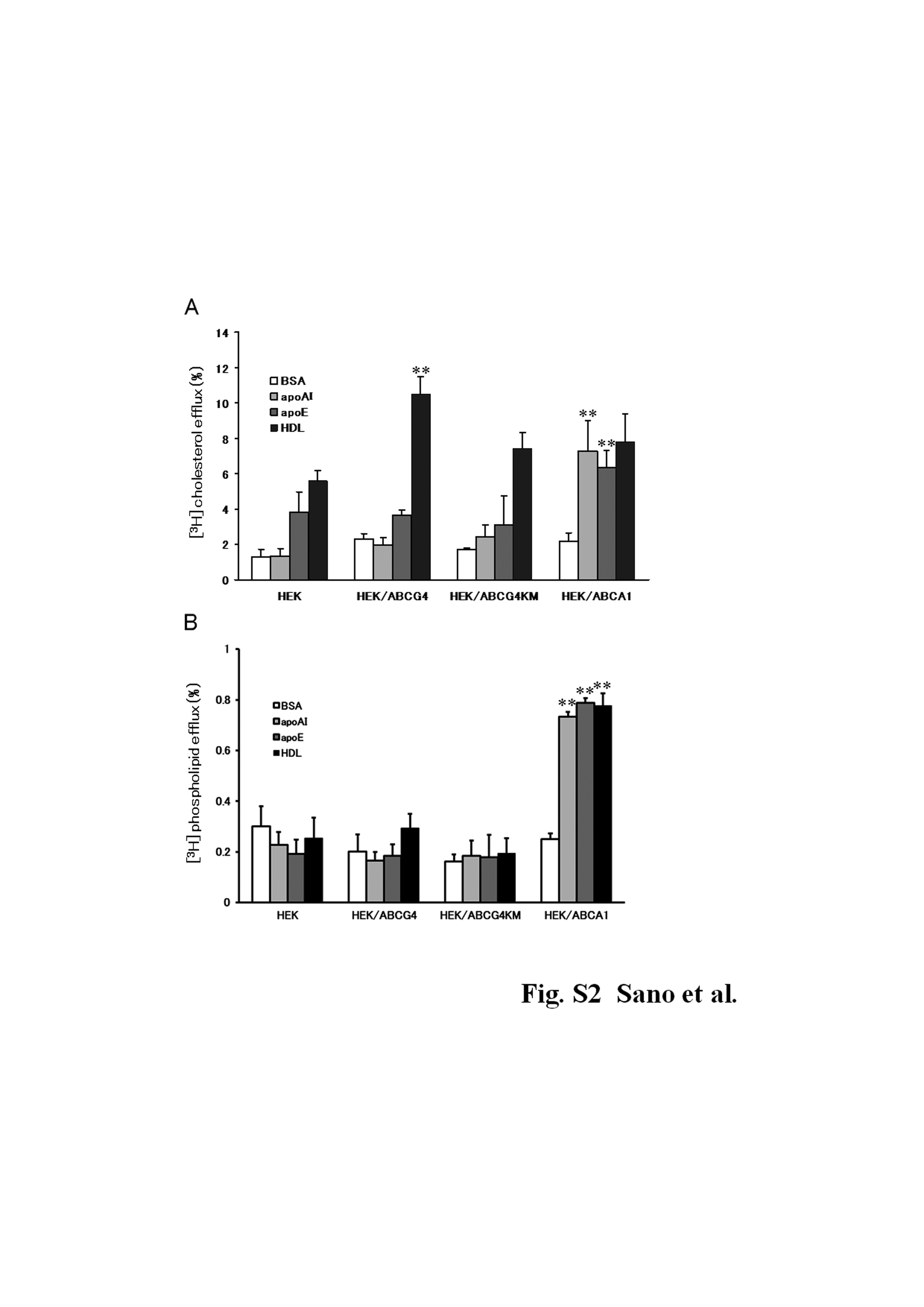

Supplement: Figure S2 — Efflux of fractional [3H]cholesterol and [3H]choline phospholipids by ABCA1 or ABCG4. Cells were labeled for 24 h with [3H]cholesterol or [3H]choline in DMEM containing 10% FBS, and the efflux of [3H]cholesterol (A) or [3H]choline phospholipids (B) from HEK293, HEK/ABCG4, HEK/ABCG4-KM, or HEK/ABCA1 cells during 4 h in the presence of 0.02% BSA alone (white bars), 0.02% BSA plus 10 µg/ml apoA-I (light gray bars), 0.02% BSA plus 10 µg/ml apoE (dark gray bars), or 0.02% BSA plus 20 µg/ml HDL (black bars) was analyzed. Average values of three experiments are presented with the SD. *P<0.05; **P<0.01, significantly different from HEK293 cells. (TIF) [file pone.0109886.s002.tif]

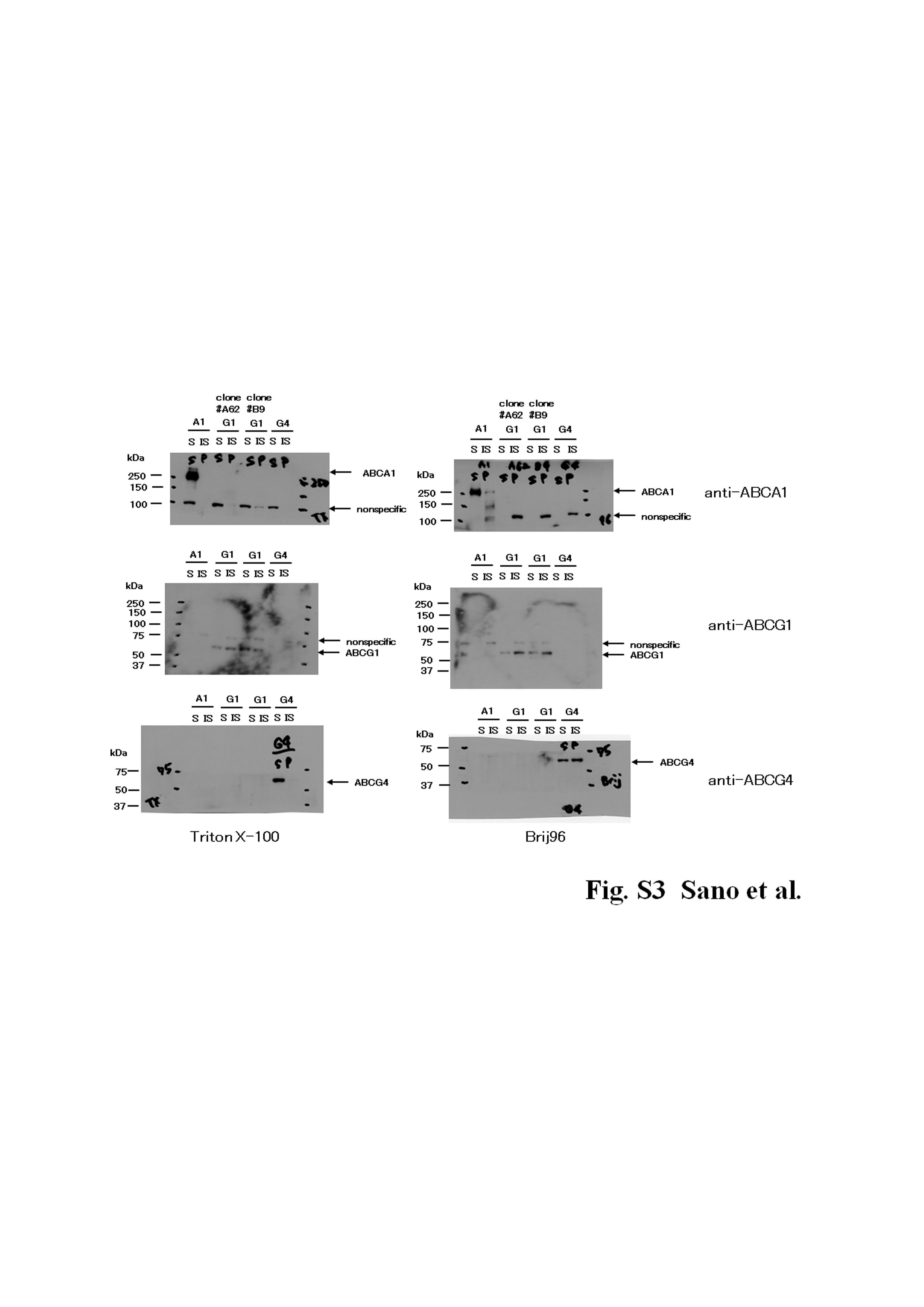

Supplement: Figure S3 — Solubility of ABCA1, ABCG1, and ABCG4 treated with Triton X-100 and Brij 96. The raw data for Fig. 2 are shown. HEK293, HEK/ABCA1, HEK/ABCG1 (clone #A62), HEK/ABCG1 (clone #B9), or HEK/ABCG4 cells were treated with 1% Triton X-100 or Brij 96, and separated to soluble (S) and insoluble (IS) fractions by centrifugation. (TIF) [file pone.0109886.s003.tif]
